# Structural and compositional characteristics of Fukushima release particulate material from Units 1 and 3 elucidates release mechanisms, accident chronology and future decommissioning strategy

**DOI:** 10.1038/s41598-020-79169-2

**Published:** 2020-12-16

**Authors:** Peter G. Martin, Christopher P. Jones, Stuart Bartlett, Konstantin Ignatyev, Dave Megson-Smith, Yukihiko Satou, Silvia Cipiccia, Darren J. Batey, Christoph Rau, Keisuke Sueki, Tatsuya Ishii, Junya Igarashi, Kazuhiko Ninomiya, Atsushi Shinohara, Alison Rust, Thomas B. Scott

**Affiliations:** 1 Interface Analysis Centre, School of Physics, University of Bristol, Bristol, BS8 1TL UK; 2 Diamond Light Source, Harwell Science and Innovation Campus, Didcot, Oxfordshire, OX11 0DE UK; 3 Collaborative Laboratories for Advanced Decommissioning Science (CLADS), Japan Atomic Energy Agency (JAEA), Tomioka-Machi, Futaba-gun, Fukushima, 979-1151 Japan; 4 Graduate School of Pure and Applied Sciences, University of Tsukuba, 1-1-1 Tennodai, Tsukuba, Ibaraki 305-8577 Japan; 5 Graduate School of Science, Osaka University, 1-1 Machikaneyama, Toyonaka, Osaka 560-0043 Japan; 6 School of Earth Sciences, Wills Memorial Building, University of Bristol, Bristol, BS8 1RJ UK

**Keywords:** Environmental impact, Environmental sciences, Energy science and technology, Materials science, Physics

## Abstract

The structural form and elemental distribution of material originating from different Fukushima Daiichi Nuclear Power Plant reactors (Units 1 and 3) is hereby examined to elucidate their contrasting release dynamics and the current in-reactor conditions to influence future decommissioning challenges. Complimentary computed X-ray absorption tomography and X-ray fluorescence data show that the two suites of Si-based material sourced from the different reactor Units have contrasting internal structure and compositional distribution. The known event and condition chronology correlate with the observed internal and external structures of the particulates examined, which suggest that Unit 1 ejecta material sustained a greater degree of melting than that likely derived from reactor Unit 3. In particular, we attribute the near-spherical shape of Unit 1 ejecta and their internal voids to there being sufficient time for surface tension to round these objects before the hot (and so relatively low viscosity) silicate melt cooled to form glass. In contrast, a more complex internal form associated with the sub-mm particulates invoked to originate from Unit 3 suggest a lower peak temperature, over a longer duration. Using volcanic analogues, we consider the structural form of this material and how it relates to its environmental particulate stability and the bulk removal of residual materials from the damaged reactors. We conclude that the brittle and angular Unit 3 particulate are more susceptible to further fragmentation and particulate generation hazard than the round, higher-strength, more homogenous Unit 1 material.

## Introduction

In March 2011, the Fukushima Daiichi Nuclear Power Plant (FNDPP) was inundated with a 15 m high tsunami following the M_W_ 9.0 earthquake that occurred off the countries eastern coast^1^. In the days that followed, a vast amount of radioactive material, in varying forms (including aerosol, particulate and gaseous species) was emitted into the environment—both locally as well as globally. The total summed source-term inventory of the individual radionuclides (excluding volatile noble gases) released from the FDNPP is approximated to 520 PBq (340–800 PBq, upper and lower bounds, respectively) or 10% of the total inventory emitted in 1986 following the accident from the Chernobyl Nuclear Power Plant (ChNPP)^2,3^. Although less radiation was released, the accident at the FDNPP was similarly rated at Level 7 (the most severe) on the International Atomic Energy Agency (IAEA) International Nuclear Event Scale (INES)—a measure of the global ramifications of the world’s second worst ever nuclear accident^4^. In contrast to the single reactor associated with the incident at the ChNPP, this high event severity at the FDNPP is a direct consequence of the multiple reactor Units involved—with contamination releases arising from three of the six boiling water reactor (BWR) Units^5^.

At the time of the accident, reactor Units 1, 2 and 3 (which entered commercial power generation in 1971, 1974 and 1976, respectively) were in power generation mode. However, following the detection of the first seismic activity, each of these reactors successfully entered an emergency shutdown (or ‘SCRAM’) through the as-standard insertion of neutron-absorbing and fission inhibiting control rods^3,6^. Attached to Unit 3, via shared pipework and ducting, the neighboring reactor Unit 4 (which became operational in 1978) was undergoing planned maintenance on 11th March 2011^7^; with the inventory of 1331 fuel elements having been moved to its own dedicated spent fuel storage pond (FSP—a deep water-filled recirculated cooling facility) located above the reactors primary containment vessel (PCV), which itself houses the main reactor pressure vessel (RPV) where the fuel is located during operation^8^. As a consequence of the recent transfer of still ‘thermally hot’ (as well as highly radioactive) fuel into the reactor Unit 4 FSP, a notable cooling requirement still existed on the material within the ponds^9^. In contrast, the sites remaining two (and most recently constructed) reactors, Units 5 and 6 (operational in 1978 and 1979, respectively), were in a period of extended ‘cold’ shutdown for planned maintenance at the time of the earthquake and tsunami; with the fuel assemblies still inside each of the RPVs (548 and 764, respectively), in addition to a greater number stored in each of the above-reactor FSP facilities (946 and 876, respectively). Owing to the time that reactors 5 and 6 had been placed in shut-down, the thermal output from these partially used (or ‘burnt’) fuel assemblies was greatly diminished, such that a significant cooling provision to the FSP was no longer required^3^. Stemming from the time difference in the construction of each of the BWR reactor Units, alongside increasing electrical output, a number of design and system variations are evident between Unit 1 and Unit 6. The most noticeable of which is the transition from Mark-I to Mark-II designs of the PCV^3^—from the classic ‘inverted lightbulb’ (torus) shape to the more structurally robust and resilient design with greater suppression pool/chamber capacity.

While each of the operational Units (1, 2 and 3) successfully ceased further reactivity (through a cessation of neutron generation) and power production, there still remained a significant amount of residual heat within each RPV that required removal in order to protect the integrity of the reactor and the fuel within. Following the detection of seismic activity and reactor shut-down, core cooling was sufficiently facilitated by the suite of in-built safety features; including the reactor core isolation cooling (RCIC) and a number of emergency core-cooling systems (ECCS). However, after the destruction of the power supplies that facilitated this heat removal (both mains electrical and back-up diesel) by the ensuing 15 m high tsunami (that arrived 30 min after the earthquake), it became impossible to suppress the rapidly rising temperatures of each core and cool the highly radioactive reactor fuel assemblies. Consequently, there resulted a series of loss of coolant incidents (LOCI) as the temperature within each RPV exceeded the melting point of the fuel (UO_2_ in Units 1 and 2; UO_2_ and a U/Pu mixed oxide (MOX) in Unit 3) and other reactor components^5^. This resulted in the structural failure, and ‘slumping’, of the reactor fuel assemblies in each of the three reactors (albeit to differing degrees)^10–12^. So extensive and at such high temperatures was this molten core composition (comprising the oxide fuel, reactor components and structural metals/concrete) ‘Corium’ mass, that following its melting through of the stainless-steel RPV, the flowing mass continued downwards onto the underlying concrete pedestal installed within the PCV—resulting in highly complex molten core-concrete interactions (MCCI)^13^.

In contrast to the downwards transition of super-heated core material within the reactor cores, which has been hard to physically visualize its extent within the RPV owing to its highly radioactive nature, the external radioactive releases that occurred at the three reactor Units (and Unit 4 FSP) were highly visual and/or detectable. The releases from reactor Units 1 and 3 were associated with large hydrogen explosions; both of which destroyed the reactor building superstructures. However, the most widespread and largest inventory activity release resulted from the non-explosive discharge from reactor Unit 2—invoked to have resulted from a breach in the structural integrity of the PCV (following extensive, later inspections using both manned entries and robotic surveillance platforms)^3,5,14^.

The ability to provenance each of the discrete radioactive release events and associated particulate material derived from each reactor Unit is a consequence of the differing operational histories, fuel characteristics and burn-ups of the fuel contained within each of the sites boiling water reactors^15^. Alongside subtle differences in the low levels of enrichment of the UO_2_ fuel (across all damaged reactor Units) and the incorporation of a partial core MOX loading (within reactor Unit 3)^7^, the primary characteristic for fingerprinting each of the reactors is the ^134^Cs/^137^Cs (radiocaesium) activity ratio (amongst other activity and isotope ratios). A summary of this Cs activity variation is illustrated in Figure S1 for the operational reactors and FSPs. These values were derived using ORIGEN simulations for the bulk ‘whole inventory’ of fuel (core or FSP) within each setting, and while variations in ratios such as the ^134^Cs/^137^Cs activity ratio are observed down the vertical length of a used fuel element (greatest towards the base owing to the increased neutron fluxes) and also its position in the reactor core itself (highest at the center, again owing to increased neutron fluxes and resultant burn-up)^16^, a widely accepted single representative value for each reactor Unit/FSP has been calculated^15^. It is such values of < 1.0 (Unit 1), circa. 1.08 (Unit 2) and circa. 1.04 (Unit 3) that have resulted in the primary 35 km north-west trending contamination plume being attributed to reactor Unit 2—with plumes of smaller spatial extent that surround the plant ascribed to the releases of reactor Units 1 and 3^17–19^.

**Figure 1:**
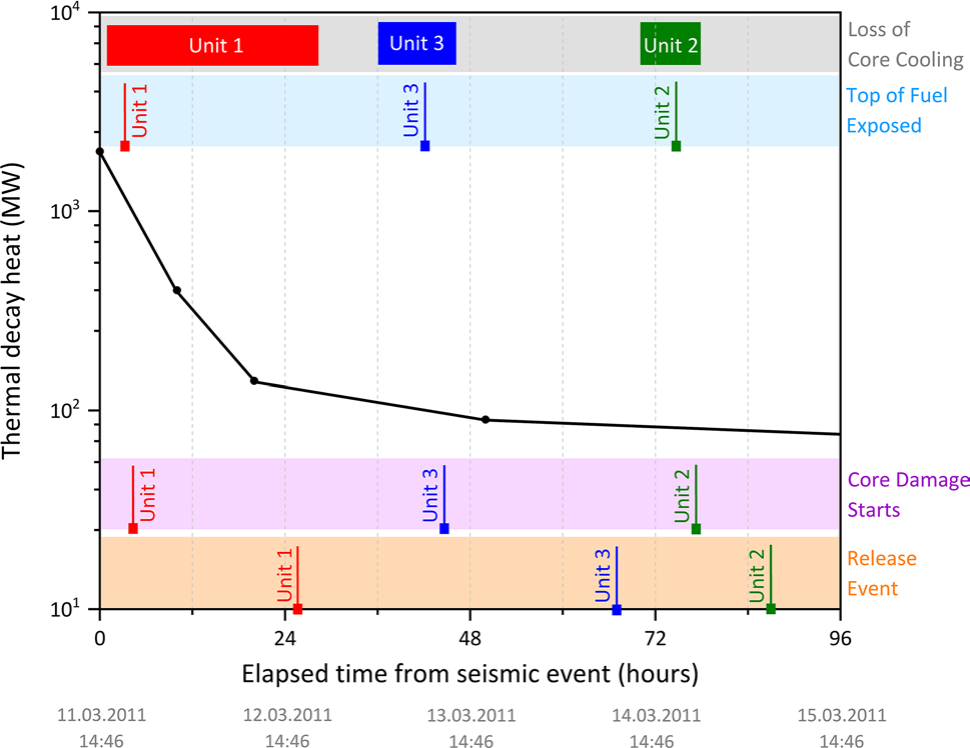
Modelled post-SCRAM reactor (thermal) decay heat associated with a typical FDNPP BWR Unit. The bars indicate periods over which appropriate core cooling capability was degraded; with the vertical lines signifying the times at which the fuel was first exposed, core damage ensued, and release events subsequently occurred from each reactor Unit^5,24^.

As well as the indicative bulk radiocaesium (activity) ratios for each of reactor Unit (and FSP), all of the FDNPP reactors are further characterized by a unique cooling chronology over the duration of the accident; a schematic of which is shown in Fig. 1. Alongside the residual thermal decay heat within each reactor core following its SCRAM and control rod insertion, illustrated in Fig. 1 are the periods over which sustained interruptions in core cooling occurred, the believed time at while both fuel was exposed and core damage was sustained, as well as the timings of release events arising from these LOCI^5^.

In contrast to earlier works that studied the fine-scale and highly spherical (~ 2 µm diameter) release material from reactor Unit 2^20–22^, this work examines and compares the larger diameter (> 100 µm) radioactive particulates emitted from reactor Unit 1 to those invoked to derive from reactor Unit 3. While both releases were the result of violent hydrogen gas accumulation explosions^3^; as is shown in Fig. 1, the emission events were separated by a period of 43 h. Owing to the greater sustained interruption in required core cooling provision and elevated level of decay heat accumulation in reactor Unit 1 in comparison to that of reactor Unit 3 (circa. 24 h, opposed to approximately 6 h), the damage sustained by the Unit 1 fuel and RPV is more significant than that of Unit 3^5^. However, despite the periodic venting of the reactors (principally into their connected suppression chambers within the Mark-I design PCV), the pressure build-up within Unit 3 was for a greater duration than for Unit 1—with core melting (albeit at a lower temperature due to the greater extent of core cooling) at a reduced rate and to a lesser extent^23^.

A reactor Unit origin nomenclature of FDNPP release particulates (based on; size, exterior form, ^134^Cs/^137^Cs activity ratio, ^135^Cs/^137^Cs atomic (isotopic) ratio, radiocaesium concentration and geographical sampling location) was first proposed by Satou et al.^25^ and has since been widely adopted following the extensive analysis of such FDNPP-derived material. Like the radioactive particulates released into the environment from the 1986 Chernobyl accident^26^, this work defined two distinct groupings of material; ‘Type A’ and ‘Type B’—a review of which is provided Igarashi et al.^22^. Such ‘Type A’ particulates are highly spherical, and resulting from their ^134^Cs/^137^Cs activity signature being analogous to the modelled core inventory^15^, are attributed to have been derived reactor Unit 2. Due to their small diameter and readily transported nature; entrained within buoyant atmospheric air masses, they have been isolated using high-volume aerosol samplers at locations over 170 km from the FDNPP site^27^, with a subset of these “Cs-balls” identified as containing reactor core composition U within their cores^20,21^. With the internal Cs component distributed in some instances homogeneously^21^ and others heterogeneously (concentrated within the particulates outermost ‘rind’)^28^, the specific activity (activity per unit volume) of this micron-scale ‘Type A’ material is significantly greater than that of the larger ‘Type B’ particulates^25^. Such ‘Type B’ material (of contrasting size, radiocaesium activity ratio and spatial distribution surrounding the FDNPP) has conversely been ascribed to reactor Unit 1; exhibiting a highly irregular surface form, 70–400 µm largest dimension and strongly near-plant occurrence.

The absence of a radioactive release particulate attributable to Unit 3 (or, by the nomenclature implemented by Satou et al.^25^, a ‘Type C’) had been undocumented ahead of the studies of both Zhang et al.^29^ and Igarashi et al.^23^. Alongside their complimentary Sr and Pu signatures analysis, respectively, the studies examined ‘dust particles’ (DP)—provenanced to Unit 1, in addition to a new form of isolated ‘soil particle’ (SP). Resulting from their dissimilar form, compositional and isotopic signatures (including a comparison to ^137^Cs/^90^Sr activity ratios of particulates with known reactor origin), these studies both invoked such (‘SP’) material to have resulted from a new emission source—reactor Unit 3, and a ‘Type C’ particulate material.

This work follows earlier studies on Unit 1 derived materials^30,31^ by evaluating the source and formational setting associated with this new inventory of Unit 3 (or ‘Type C’) FDNPP-sourced particulate. Alongside elucidating the current in-reactor conditions, we consider the likely environmental implications of this new form of ejecta particulate by evaluating its likelihood to breakdown, mobilize and introduce subsequent contamination hazards within the dynamic Fukushima environment close to the plant. In this work, laboratory X-ray tomography (XRT) combined with synchrotron radiation (SR) XRT and X-ray fluorescence (XRF) analysis is applied to a suite of Unit 3 derived particulates—the results of which are compared to formerly examined Unit 1 material^31^, applying analogues associated with volcanic ejecta (pyroclasts).

## Results

### Gamma-ray spectroscopy

The results of gamma-ray spectroscopy on each of the release particulates to derive the ^134^Cs/^137^Cs activity ratios and consequently to confirm the FDNPP reactor responsible for its release, are shown in Table 1, all of which are decay corrected to 11th March 2011. From Table 1, the disparity between the radiocaesium activity ratios of the two sample suites (CF-xx and OH-xx) is apparent, with a ^134^Cs/^137^Cs activity ratio of < 1 (analogous to Unit 1^15^) determined for the CF-xx particulates—obtained from a region to the north-west of the plant through which low-altitude aerial mapping had formerly attributed a similar (Unit 1) provenance to the contamination^18,19^. This CF-xx particulates ^134^Cs/^137^Cs activity ratio is in direct contrast to that of the OH-xx inventory of material, all of which possess a ^134^Cs/^137^Cs activity ratio of > 1 (Table 1 and Figure S1). Such a particulate radiocaesium signature matches the environmental signature of the contaminated Okuma region (to the south-west of the FDNPP site) from which the material was sourced^18,19^, attributed to have been contaminated by the reactor Unit 3 hydrogen explosion on 14th March 2011.

**Table 1:** Summary of ^134^Cs/^137^Cs activity ratios (derived by gamma-ray spectroscopy) and the associated measurement uncertainties (2σ) for the representative particulates studied in this work, alongside the attributed FDNPP reactor Unit. *Decay corrected to March 2011.

Particle reference	^134^Cs + ^137^Cs activity ± 2σ (kBq)*	^134^Cs/^137^Cs activity ratio* (± 2σ)	FDNPP reactor unit
CF-01-1	37.2 ± 0.62	0.93 ± 0.02	1
CF-01-T18	0.22 ± 0.02	0.94 ± 0.03	1
CF-01-R024	10.6 ± 0.50	0.94 ± 0.02	1
CF-01-T06	0.36 ± 0.03	0.93 ± 0.03	1
CF-01-T009	14.2 ± 0.44	0.92 ± 0.02	1
OH-06-07	0.17 ± 0.02	1.03 ± 0.03	3
OH-06-10	0.08 ± 0.01	1.03 ± 0.04	3
OH-02-01	3.16 ± 3.16	1.05 ± 0.03	3
OH-02-01	2.18 ± 2.18	1.04 ± 0.03	3

### External particle morphology and composition

**Figure 2:**
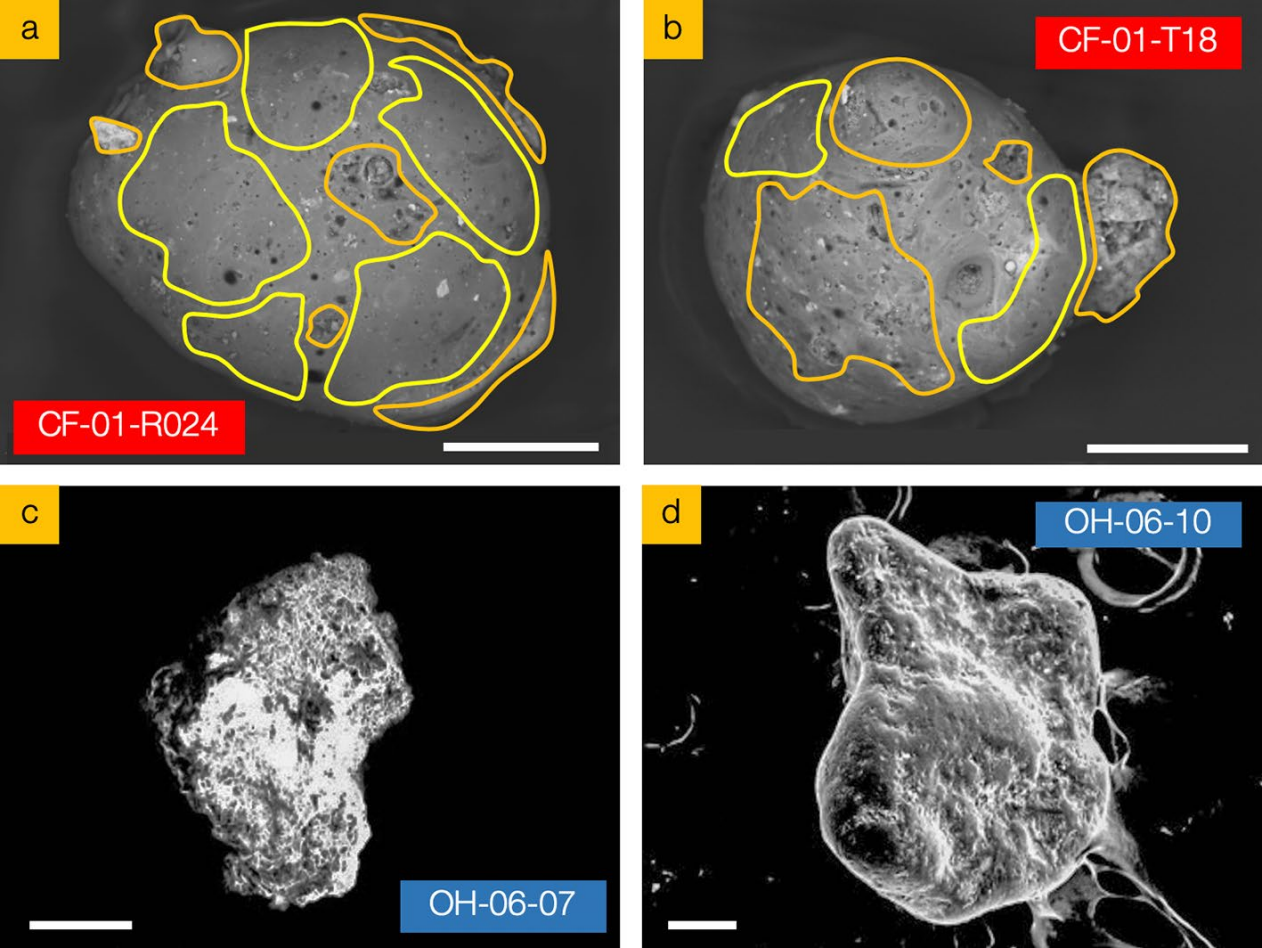
Electron microscope images of representative FDNPP release particulate; (**a**,**b**) reactor Unit 1 (CF-01-R024 and CF-01-T18) and (**c**,**d**) reactor Unit 3 (OH-06-07 and OH-06-10). Scale bars = 100 µm.

Electron microscope images detailing the surface morphology of representative particulates from both reactor Unit suites are presented in Fig. 2a–d; selected from a suite of isolated sub-mm samples possessing characteristic features (both surface and internal), elemental composition and isotopic activities—all isolated from bulk sediment samples collected from the same geographical location. As observed during earlier studies on Unit 1 derived samples^31^, the exterior surface of such FDNPP material is highly variable: sub-regions of the particulate are well-rounded and smooth in form (highlighted yellow in Fig. 2a,b), which markedly contrasts with other areas where the surface is constituted by highly angular and protruding material of a fragmented and assimilated nature (highlighted orange in Fig. 2a,b). The fibrous characteristics of a number of these surface manifestations are consistent with those formerly observed across the exterior of other Unit 1 release particulates examined^32^—attributed to result from the incorporation of Si-based thermal insulation materials.

Such regions, observed across all Unit 1 derived particulates examined, are interstitial to those where surface voids/pits are observed. We calculate that these regions comprise between 8 and 15% pitting by surface area—with an average diameter of 2.8 µm, although the largest surface void possesses a diameter of 73 µm (Fig. 2b).

However, in contrast to the intra-particle variability exhibited by the Unit 1 material, the representative particulate sourced from Unit 3 is considerably less complex in surface form—the SEM images of OH-06-07 and OH-06-10 are shown in Fig. 2c,d. While the particulates have a textured surface, they are all also more rounded in form and with no angular extrusions or surface pits/voids. Across all such Unit 3 particles, we observe no fibrous morphologies, as observed associated with the surfaces of the Unit 1 FDNPP-derived samples.

**Figure 3:**
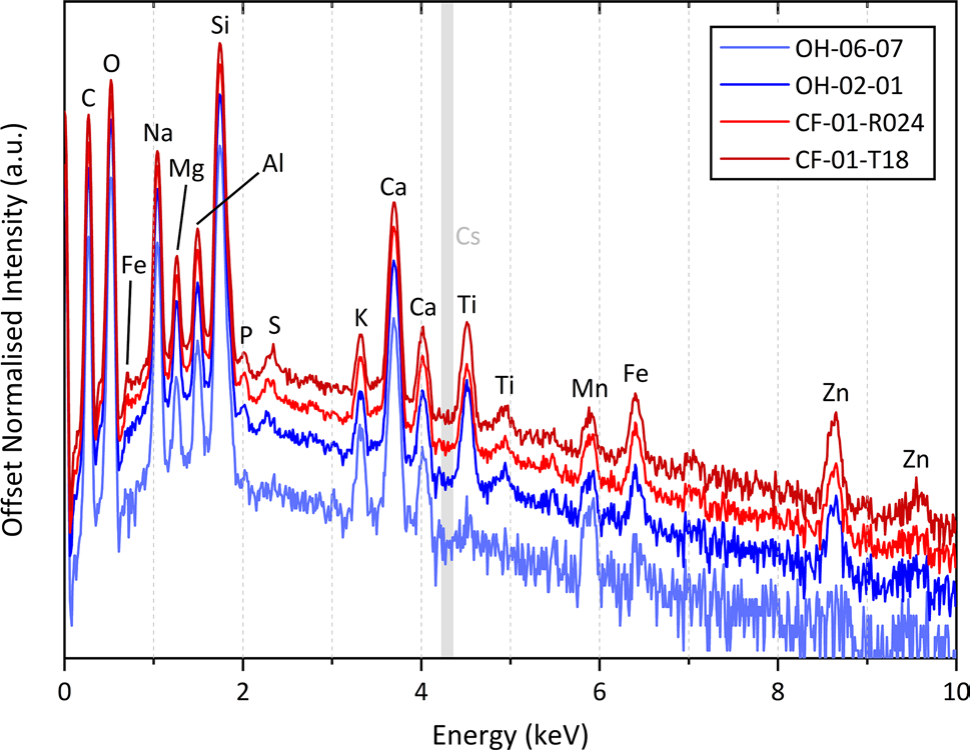
EDS compositional analysis of representative release particulates from FDNPP reactor Unit 1 (red) and Unit 3 (blue).

The surface morphological analysis via electron microscopy is supported by the co-incident compositional analysis provided by bulk (whole) particle EDS, with the results of this analysis on two representative Unit 1 (CF-01-T18 and CF-01-R024) and two representative Unit 3 (OH-06-10 and OH-06-07) particulates presented in Fig. 3. As determined in earlier works^25,33^, the primary constituents of the Unit 1 release material (red in Fig. 3) are Si, O, C, Na, Ca and Al—with the Unit 3 material (blue in Fig. 3) also comprising the same primary elemental constituents. Further elements (albeit with less abundance) common between both particulate suites, whose occurrence is also consistent with these earlier studies include Mg, Al, K, S, P, Mn, Fe, Zn and Ti. The work by Ono et al.^33^ also detected Pb and Cr in some Unit 1 derived particles, however, none of these elements were found by EDS analysis to exist within any of the Unit 1 or Unit 3 particle samples examined within this work.

This compositional similarity between Unit 1 and Unit 3 particulate is also shared with the smaller, and highly spherical, material derived from reactor Unit 2^20,27^, however, a fundamental difference between such significantly larger (Unit 1 and Unit 3) particulates and the micron-scale (Unit 2) ‘Cs-balls’ is the detectable occurrence of Cs via EDS analysis (where limits of detection are ~ 0.1 wt%). As determined during earlier studies that examined the Cs abundance within reactor Unit 1 particulate^31^, the ‘per particle’ activity, and therefore the concentration of radiocaesium within such Units 1 and 3 particles is more than 4 orders of magnitude less per unit volume than the smaller Unit 2 material^22,25^, and is highly concentrated within the material—rather than being associated with the material’s spherical outer surface. Such a low abundance, combined with the concentrated nature within the similar Unit 1 material, therefore elucidates as to the inability to detect Cs within such Unit 3 material via EDS.

### Internal particle structure

**Figure 4:**
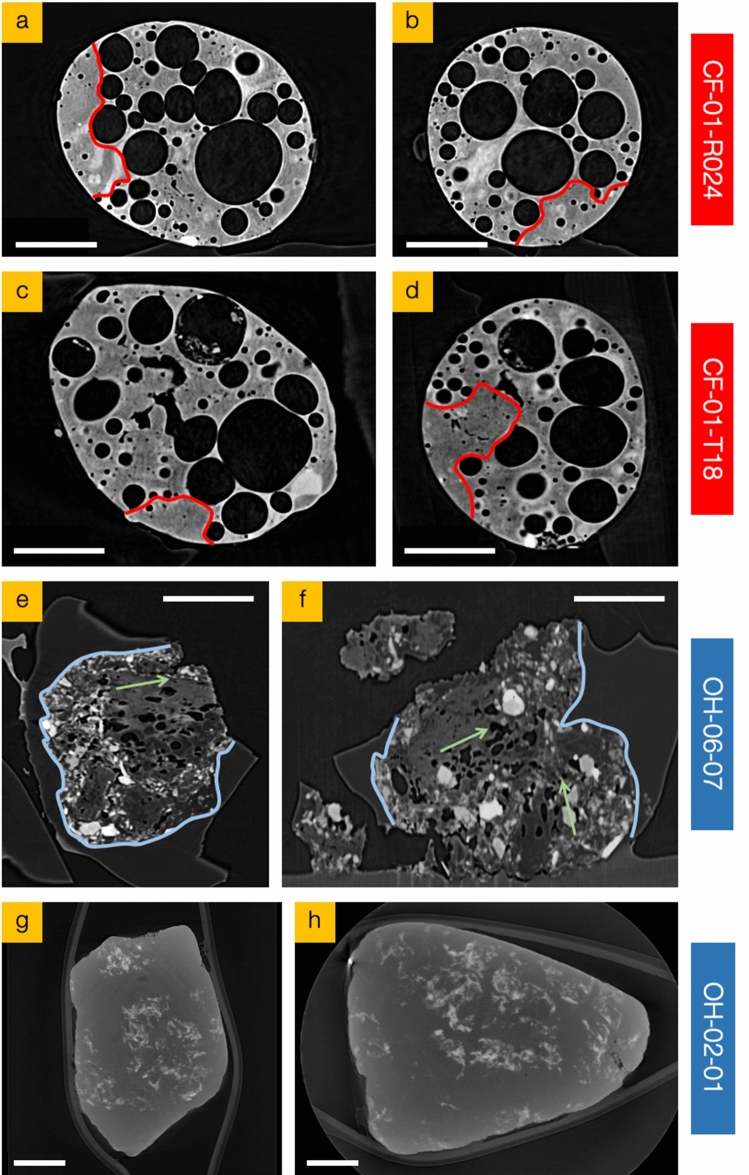
Laboratory X-ray tomography sections obtained at perpendicular orientations through the both central horizontal and vertical planes of two of the representative particulates from each suite; (**a**,**b**) CF-01-R024 (Unit 1), (**c**,**d**) CF-01-T18 (Unit 1) and (**e**,**h**) OH-06-07 (Unit 3), (**g**,**h**) OH-02-01 (Unit 3). Regions absent of voids within the representative Unit 1 particulates are red in (**a**)–(**d**). Scale bars = 100 µm.

Absorption contrast XRT sections obtained at perpendicular orientations through the central axes of representative Unit 1 and Unit 3 particulates are shown in Fig. 4. Observable from these tomographic slices is the contrasting internal form of the two suites of reactor-sourced particulate—as well as the previously described external particulate form and morphology. As detailed in the earlier work of Martin et al.^31^ that examined Unit 1 material, and as shown in Fig. 4a–d for a subset of representative Unit 1 particulates (CF-01-R024 and CF-01-T18), the internal volume is characterized by a significant internal void volume—comprised of a bimodal distribution of nearly spherical bubbles. With the largest void of circa 300 µm diameter, the larger of the bimodal void groups (concentrated within the central region of the particulates) has a mean diameter of 71 µm, whereas the smaller void grouping (located closer to the particles circumference) possesses a mean diameter of 17 µm. The total internal porosity (from both small and large voids) we calculate for the CF-xx suite of Unit 1 particulates is 27% (range 24–31%), distributed throughout the majority of the total internal volume. However, all such Unit 1 derived particulates contain regions deficient of voids—as marked (red) in Fig. 4; herein defined as the interconnected internal volume for which the porosity is below 2%. These void-depleted regions occur in areas of identical (Si-based) composition to the remainder of the particle—consistent with the EDS elemental results shown in Fig. 3.

The internal forms of the Unit 3 ejecta material, revealed by the orthogonal XRT sections of the two representative particles shown in Fig. 4e–h are markedly different from the Unit 1 ejecta characterized by the near-spherical voids. The angular OH-06-07 particle (Fig. 4e,f) has a variably rough surface texture and shows a highly heterogeneous and varied internal structure—with angular amorphous regions (highlighted blue) enclosing a central core comprising fragment inclusions (also Si-based) and elongate/non-spherical voids, which constitute 8% total internal porosity. This particle contains regions of preferred orientation of the voids, with the long-axes of the approximately 20 µm voids aligned—illustrated in Fig. 4e,f by the green arrows.

Furthermore, the location and distribution of (non-spherical) voids within the Unit 3 sourced particulates (as illustrated in Fig. 4e,f) is different to those within the inventory of Unit 1-derived material. The voids within Unit 3 material have a unimodal size distribution and are strongly concentrated within the core regions of the particles, with 66–81% of the total void volume occurring > 100 µm from the perimeter of the particulates. This outermost void-depleted region is conversely rich in solid fragment inclusions, or as illustrated within Fig. 4e,f, is amorphous in nature.

The other representative Unit 3 particle (OH-02-01), the tomographic sections of which are shown in Fig. 4g,h, is very different from both the Unit 1 particulates shown in Fig. 4a,b and the other Unit 3 particle (OH-06-07) previously described. It is the largest particle studied, and despite having the same composition as the other samples (both Unit 1 and Unit 3), the XRT data show it is entirely solid—with no internal porosity or inclusions. In contrast to the other Unit 3 particle (OH-06-07) it has a smooth and well-rounded exterior surface—likely to result from the subsequently discussed localized in-reactor condition variations (including pressure, decay temperature and duration) and the differing locations from which the particulate material was derived; with the scale of the MCCI reaction in reactor Unit 3 being considerably smaller than that of reactor Unit 1, owing to the smaller amount of molten fuel (Corium) melt-through^12^.

### Internal void morphology

**Figure 5:**
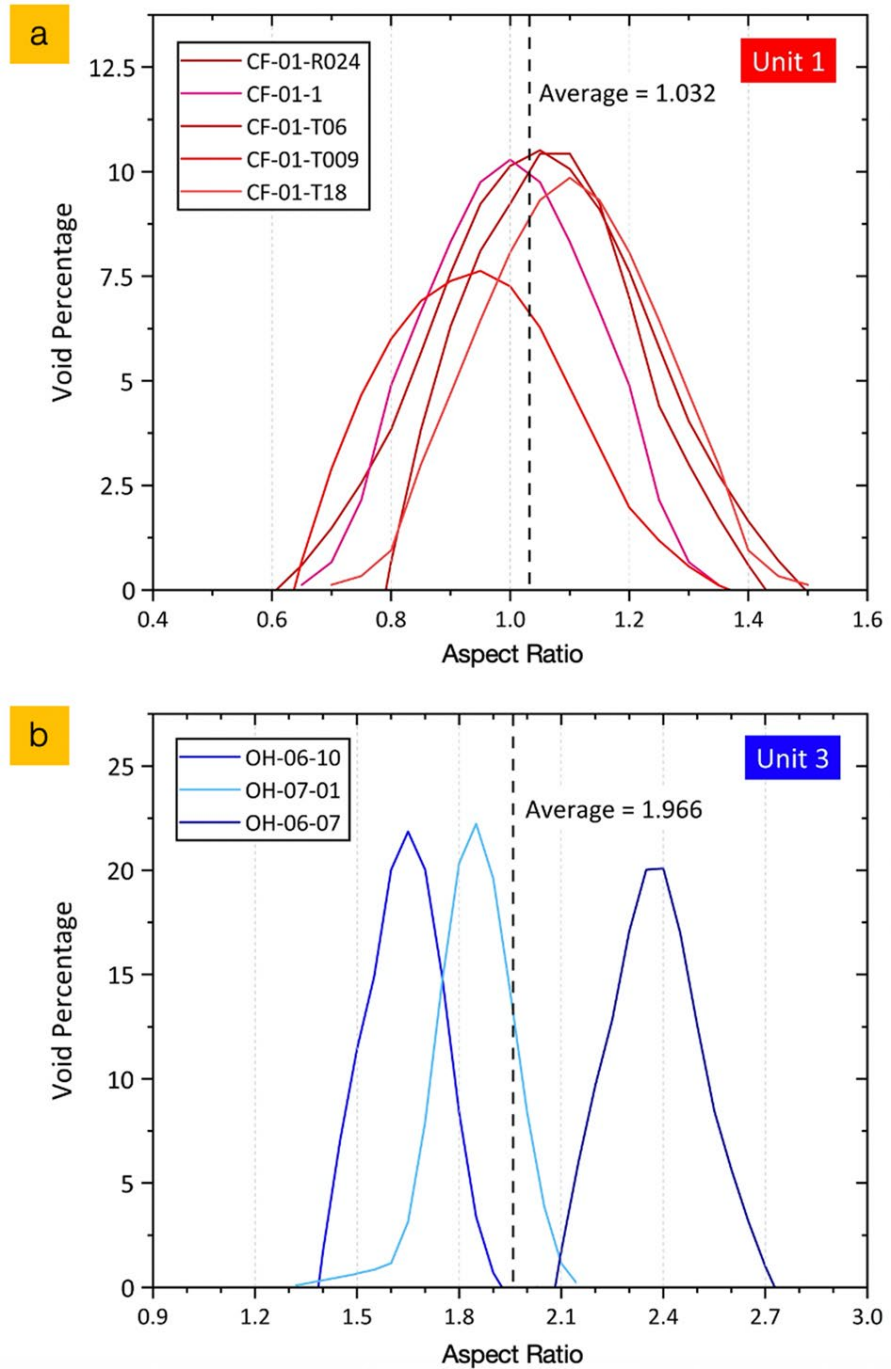
Void aspect ratio analysis of XRT sections (obtained at sequential 20 µm height increments/slice thicknesses) through; (**a**) Unit 1 particulates and (**b**) Unit 3 particulates.

The forms (shape) of voids in representative suites of Unit 1 and Unit 3 particulates were quantified with the aspect ratio (i.e. the length of longest axis divided by the dimension measured perpendicular to the long axis) measured on image slices from laboratory XRT analysis (Fig. 5). From Fig. 5a, the particulates attributed to reactor Unit 1 have an average void aspect ratio of 1.03; reflecting the near-spherical shape of the bubbles within the Si-based matrix. In contrast, the analysis on the (albeit smaller number of) voids within the Unit 3 material, as presented in Fig. 5b, confirms the highly non-spherical/rounded form of the voids evidenced through XRT analysis. For this OH-xx inventory of material, we calculate the average void aspect ratio is circa double that of the CF-xx (Unit 1) samples, at 1.97—with the bubbles approximately twice the length in one axis as the corresponding perpendicular axis. The OH-06-07 particle, with tomographic sections shown in Fig. 4e,f exhibits the greatest degree of void elongation—with a mean aspect ratio of 2.39.

### Bulk internal particle composition

**Figure 6:**
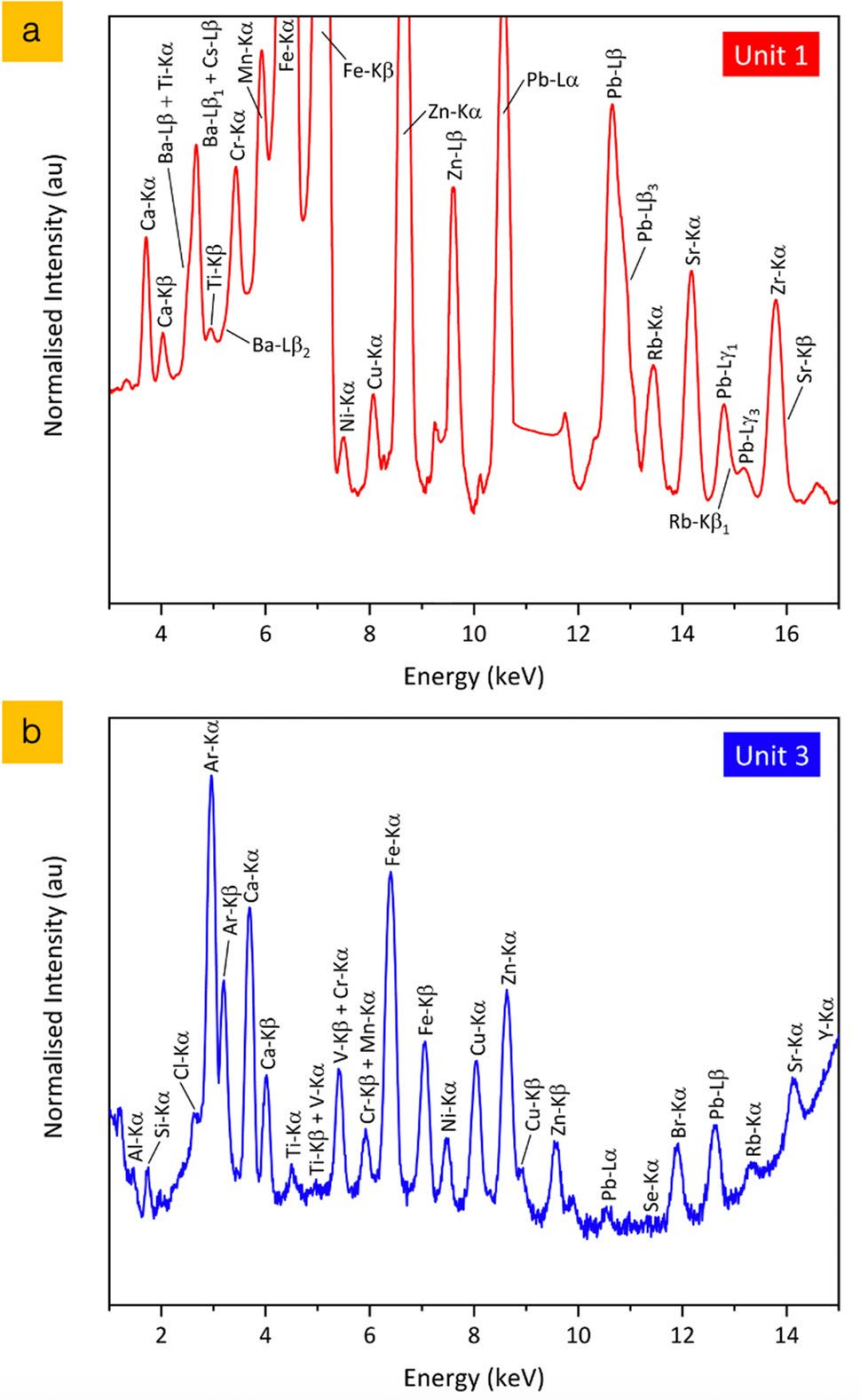
Synchrotron radiation XRF spectrum obtained from the entire volume of particles (**a**) CF-01-R024 (Unit 1), and (**b**) OH-06-07 (Unit 3).

Complementary to the surface EDS analysis results presented in Fig. 3, the application of summed SR-µ-XRF results obtained from the entire internal volume of particulates CF-01-R024 and OH-06-07, representative of the other particles within the suite, are presented in Fig. 6a,b, respectively. While the emission peaks below 3 keV of elements including C, O, S, P, K, Na, Al, Si, Cl and Mg are not measurable on the I13-1 beamline used to study the CF-01-R024 (Unit 1) particle in Fig. 6a; a lower energy range is attainable on the I18 beamline (albeit with reduced detection sensitivity over higher energies) used to analyze the OH-06-07 (Unit 3) materials. Although differing elemental concentrations/peak intensities are observed—both samples possess an equivalent overall composition, with significant Fe contribution. In contrast to the EDS analysis of the particulates, the characteristic emission peaks from the SR-µ-XRF highlights the occurrence of Pb, Cr, Cu, Ni, Rb and Sr within both these Unit 1 and Unit 3 particulates, a consequence of the enhanced detection limits of the technique.

Such a superior detection limit of SR-µ-XRF, combined with entire volumetric analysis (opposed to purely surface quantification afforded by EDS) additionally details the occurrence of Ba, Zr and trace quantities of Cs within the Unit 1 sample (Fig. 6a), with the presence of Se, Br and Y additionally found within the particulate sourced from reactor Unit 3 (Fig. 6b). The occurrence of Ar within the XRF spectrum from the OH-06-07 sample is a result of Ar fill-gas present within the experimental setup. With the exception of Se, Br, V, Ti and Y, the occurrence of these elements within FDNPP Unit 1 and Unit 3 derived particulates aligns with the results of Ono et al.^33^.

Alongside the occurrence of V, Se, Br and Y as constituents of the Unit 3 material, in contrast to the Unit 1 particulate, from the two SR-µ-XRF spectra presented in Fig. 6a,b, we observe a number of compositional differences between the two particulates of differing provenance. While Fe, Zn are Ca are major constituents of both particle types, we determine both Pb and Zr to occur at elevated concentrations in this (CF-01-R024) Unit 1 sample over the Unit 3 material—a commonality we consistently measure between the two particulate suites, produced from explosive H_2_ gas events at the two FDNPP reactors.

### Internal elemental distribution and species coincidence

**Figure 7:**
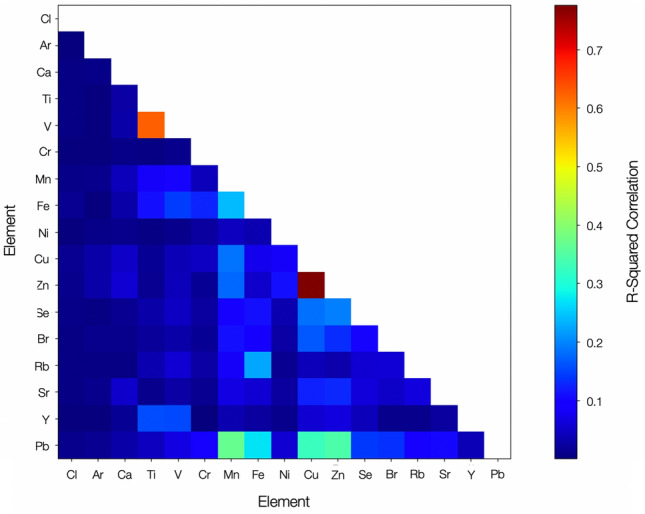
Species coincidence analysis of pixel elemental signatures determined using SR-µ-XRF mapping of the Unit 3 derived, OH-06-07, particulate.

Applying discretization to the 3D SR-µ-XRF results of the Unit 3 particulate typical of the reactor-derived inventory of material, yields the elemental correlation analysis displayed in Fig. 7, derived from the SR-µ-XRF map shown in Figure S3. From this analysis, while an absence of inter-element coincidence (via R^2^ correlation) exists for the vast majority of elements, we observe significant (> 0.3) species correlation for a number of elemental pairings; Mn–Fe, Pb–Fe, Pb–Cu and Zn–Pb, with greatest correlation (> 0.6) between Zn–Cu and Ti–V. Albeit with a degree of variability, we determine these species pairings as representative for the Unit 3 material examined.

**Figure 8:**
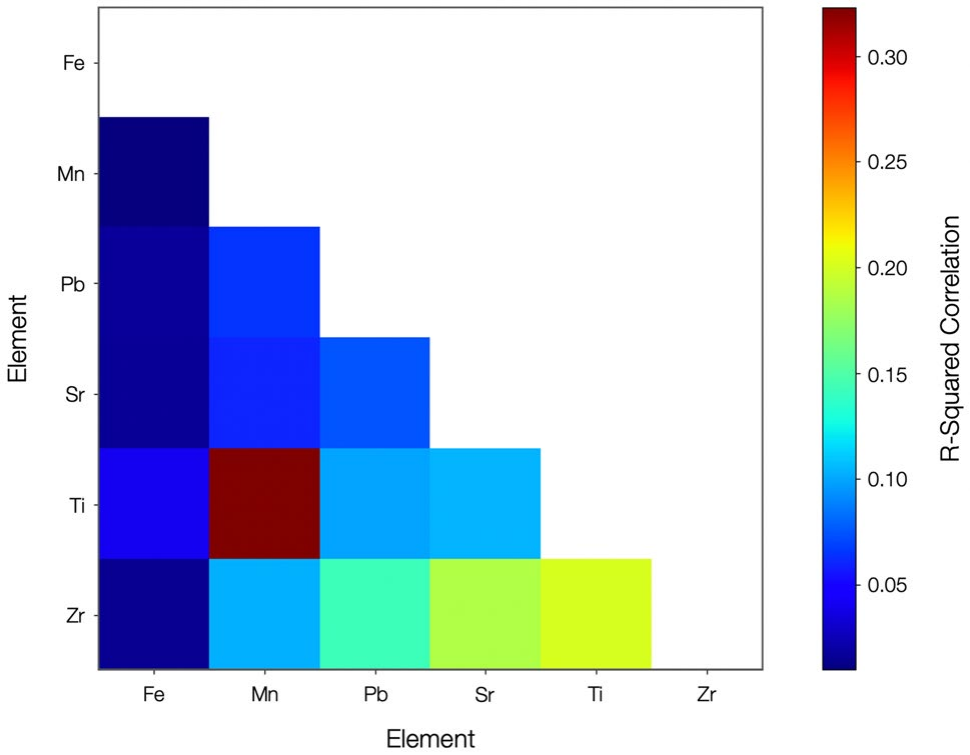
Species coincidence analysis of pixel elemental signatures determined using SR-µ-XRF mapping of the Unit 1 derived, CF-01-R024, particulate.

Such a high degree of species co-incidence between a number of elements is in contrast to the inter-elemental distribution we observe for Unit 1 particulate the results from a subset of the largest comparison are presented in Fig. 8. With correlation values of < 0.1 for all other elements, we observe a much lower level of coincidence correlation—with a maximum R^2^ value of 0.32 between Mn and Ti, and values of 0.12–0.21 for Zr associated pairings. As formerly identified in earlier works studying Unit 1 particulates^31^, both Cs and Sr are similarly observed in the representative Unit 3 material to occur at enriched near bubbles.

## Discussion

With prior electron microscope analysis having identified its fibrous nature (supported by EDS compositional analysis)^32^, it is widely accepted that glass fibre insulation material is the source of the bulk Si that is the primary constituent of the particulates released following the explosive H_2_ gas explosion at reactor Unit 1. This insulation, which was used extensively around the reactor Unit, especially around the PCV, was formed through a two-step melting and extrusion process of a silicate rock precursor that produced fibers of 4–5 µm mean diameter. We invoke a similar provenance to the bulk material constituting the invoked Unit 3 particulates because their compositions are similar to those from Unit 1; with equivalent insulation used within Unit 3 (surrounding the PCV^34^) and the explosive release mechanisms also being identical.

We formerly stipulated the likely formational processes responsible for the Unit 1 particulates^31^, concluding that their intricate internal structure is the consequence of the complex high-temperature and high-pressure in-reactor environment. This material was emitted during the first of the release events on the 12th March 2011, while the reactor existed at the greatest temperatures following the complete loss of core cooling provision (Fig. 1).

Through an assessment of the elemental and isotopic constituents and ratios, Igarashi et al.^23^ concluded that owing to variations in ratios of refractory (^239+240^Pu) to volatile (^137^Cs) species, reactor Unit 1 reached the greater temperatures at the time of radioactive particulate formation than either of Unit 2 or 3. Satou^34^ also concluded that the temperature within Unit 1 was the greatest of the three FDNPP reactor Units from the application of empirically derived volatility differences in Ag (^110m^Ag) and Cs (^137^Cs)^35^. Such reasoning aligns with the known event history presented in Fig. 1, whereby the cooling chronology of reactor Unit 3 (prior to the eventual explosive release) is markedly different to that of reactor Unit 1—with the reactor sustaining a smaller break in cooling and a later release event (on 14th March 2011). However, the reactor was maintained at elevated temperatures and pressures for longer than Unit 1. It is this difference in core conditions and overall accident duration that we invoke to represent the mechanism through which particulates of contrasting form were derived from the two reactors.

While the composition of the material examined in this study, that we invoke to have been derived from Unit 3, is largely analogous to that derived from reactor Unit 1, a number of minor compositional differences are observed. Despite its radioactivity being a result of radiocaesium (^134+137^Cs), the SR-µ-XRF analysis of the OH-xx suite of particulates does not identify the occurrence of Cs within any such sample, therefore suggesting that the concentration is very low—likely distributed throughout the entire particulate volume rather than a discrete, high-concentration, occurrence.

This first measured occurrence of Se, Br and Y (using SR-µ-XRF) within Fukushima-derived (and Unit 3) particulates is a consequence of the inclusion, into the bulk silicate precursor material, of such volatilized (Se, Br) or molten (Y) fission product species over the duration at which the PCV existed at elevated temperatures and pressures—allowing their diffusion and amalgamation. The combination of the non-detection of such species via EDS—requiring SR analysis to identify their existence, signifies the low abundance of such fission product elements within the bulk particulates. A radiogenically stable or long-lived nature of the Y, Br and Se accounts for their absence when such particulate was subjected to gamma-ray spectroscopy analysis despite the significant counting times. Therefore, these elements are most likely the isotopes of ^89^Y (stable); ^81^Br (stable); alongside various Se; ^77^Se (stable), ^78^Se (stable), ^79^Se (t_1/2_ = 10^5^ years), ^80^Se (stable), ^82^Se (t_1/2_ = 10^20^ years).

The compositional coincidences recorded within the Unit 3 particulate likely result from the elemental associations of the precursor contributing materials—remaining associated with one another during the subsequent melt process. Regions of Ti–V and Fe–Mn arise from alloying additions within steels; whereas the Cu–Zn pairing having been input into the particulates from electrical components, wiring, or the possible existence of brass components. The high degree of correlation between the number of Pb-based elemental pairings (Pb + Fe, Mn and Zn) could represent the combination of the once extensive volume of Pb shielding with the constituents of the RPV/PCV stainless steel superstructure [either primary (Fe), alloying elements (Mn, Zn), or anti-corrosion coatings (Zn)]. The occurrence of Pb-Cu coincidence is similarly the result of Pb sourced from the shielding materials—combined with Cu derived from electronics, wiring, or potentially, brass components.

Through their assessment of the reactor core temperatures (at the time of the radionuclide releases), Pu isotope mass spectrometry analysis and the known temperature-dependent species (elemental) release behavior under accident conditions^35^, Igarashi et al.^23^ determined that particulates derived from reactor Unit 1, formed under the highest temperature atmosphere—contained the greatest actinide abundances. This is a consequence of non-volatile radionuclides, such as U, Np and Pu, being more volatile at extreme fuel temperatures (~ 2200 °C)^36^; with the underpinning experimental studies of Pontillon et al.^37^ deriving release fractions of 10% for U and Np, while a very low value (< 1%) was measured for Pu. In this study on Unit 3 derived material, no actinide species were observed within any of the particulates (in contract those formerly examined from Unit 1^30^). The absence of such non-volatile radionuclides aligns with the conclusions of Igarashi et al.^23^ and the known in-reactor temperature conditions of Unit 3 (and Unit 2) at the time of release—with the temperature suppressed to limit the release fraction.

The suites of highly porous glassy airfall material from both reactor Units have similarities with primary volcanic particles and there may be analogous processes and dynamics involved in their evolution. In particular, as a fragment of molten material is ejected, it will rapidly decompress and cool—with this decompression resulting in expansion of any pre-existing bubbles contained within the melt while simultaneously driving the exsolution of other volatile elements. This coupled process together results in the nucleation and growth of bubbles—with additional contributions arising from the extensive gaseous volume within the physical environment surrounding the reactor^3,5^—composed of fission products, noble gases, and hydrogen^38^.

The overriding factor on the form and internal void morphology within different volcanically or FDNPP emission particulates is the viscosity, or resistance to deformation. Typical of many fluid scenarios, as the melt cools it becomes increasingly viscous, with ductile deformation ceasing as it crosses the glass transition (melt changes on cooling to a polymer glass^39^). The EDS-derived SiO_2_ wt% content of all particulates examined in this study is between 64 and 69% (average: 66%), comparable to a typical dacite magmatic melt, wherein applying the formulation of Giordano et al.^40^ yields a viscosity of 6 Pa s at 1800 °C, 9 × 10^3^ Pa s at 1200 °C, and a corresponding glass transition temperature (i.e. a viscosity of 10^12^ Pa s) at approximately 700 °C. While these values represent an appropriate estimate for the bulk silicate material of homogenous composition, as identified formerly^31^, the minor variations in the X-ray attenuation (greyscale), observed in Fig. 4, reflect heterogeneities in the materials elemental composition and physical properties. Notably, a number of bubbles within the Unit 1 particles (Fig. 4a–d), possess bright ‘halos’ (increased X-ray attenuation) around (namely larger) voids likely due to local differences in the amount of volatile (fission product) elements around their circumference. There are two potential causes of these haloes: volatile exsolution or volatile resorption. While volatiles diffuse into bubbles (e.g. as bubbles grow during decompression), volatiles can be depleted in the melt at the bubble margin; on the other hand, if the volatiles are resorbing (e.g. due to increased volatile solubility due to cooling) then there can be volatile enrichment in the melt around the bubbles^41^. As Cs and Sr are enriched near bubbles, we suggest that these haloes reflect late-stage volatile resorption as the molten particle cooled, before the volatiles were quenched in the glass. The halo may, therefore, appear bright because of the high atomic number of Cs and Sr, relative to Si.

The voids (bubbles) in the Unit 1 particulates have an overall bi-modal size distribution, but as shown in Fig. 4a–d, there is a tendency for bubbles to be smaller and the bubble/glass volume ratio to be lower towards the particle margins. From studies on the formation of volcanic analogues, there are two primary mechanisms for this bubble texture pattern: (i) different cooling rates of the margin and interior^42^, or (ii) diffusion of volatiles out of the particle inhibiting bubble growth in the margin^28^. For the cooling rate mechanism, the interior must remain at elevated temperatures for longer than the margin—with the bubbles having greater time to expand (and in some instances coalesce) in the interior. The outer surface of these particles is smooth and not fractured (Fig. 4), therefore, in this scenario, the exterior margin (although cooler than the interior) would have to remain sufficiently hot during the expansion of interior bubbles for the margin to deform viscously, rather than fracture—having passed through the glass transition. The difference in bubble texture between the interior and the margins of the Unit 1 particulates could also be attributed to diffusion of volatile species out of the hot particles leaving the melt in the outermost region depleted in volatiles species thereby locally reducing bubble nucleation and growth (i.e. volatiles near the outside leave the particle rather than diffusing into bubbles). In contrast to the haloes around interior bubbles, there is no enrichment in Cs and Sr in the glass near margins of these particles; however, there is no direct evidence of depleted volatile content of the glass at the particle margins.

A more detailed study of the distribution of species with different diffusivities in conjunction with physical modeling would be needed to be definitive on the mechanism for generating the bubble texture pattern. However, due to the lack of evidence of volatile depletion in the marginal glass, we tentatively propose that the larger bubbles in the interior are dominantly due to a greater time for expanse as the interior cooled slightly slower than the margin^21,28,31^. The particulates would have rapidly thermally equilibrated with the surrounding environment due to their small size (each with maximum dimensions of < 500 µm). This is evidenced through the timescale for cooling (τ_cool_) by conduction^43^:

$$ \tau_{cool} = \frac{{L^{2} }}{D} $$

where L is the characteristic length-scale and D is the thermal diffusivity. For example, τ_cool_ = 0.01 s for D = 10^–6^ m^2^/s and a typical particle size with L = 100 mm. Consequently, temperature gradients within the (formerly molten) particulates at the time of release would have been short-lived. The rate at which the molten particle cools (e.g. time to reach the glass transition temperature at which it becomes solid) will therefore depend strongly on the particles trajectory and the temperature of the environment into which it is ejected.

**Figure 9:**
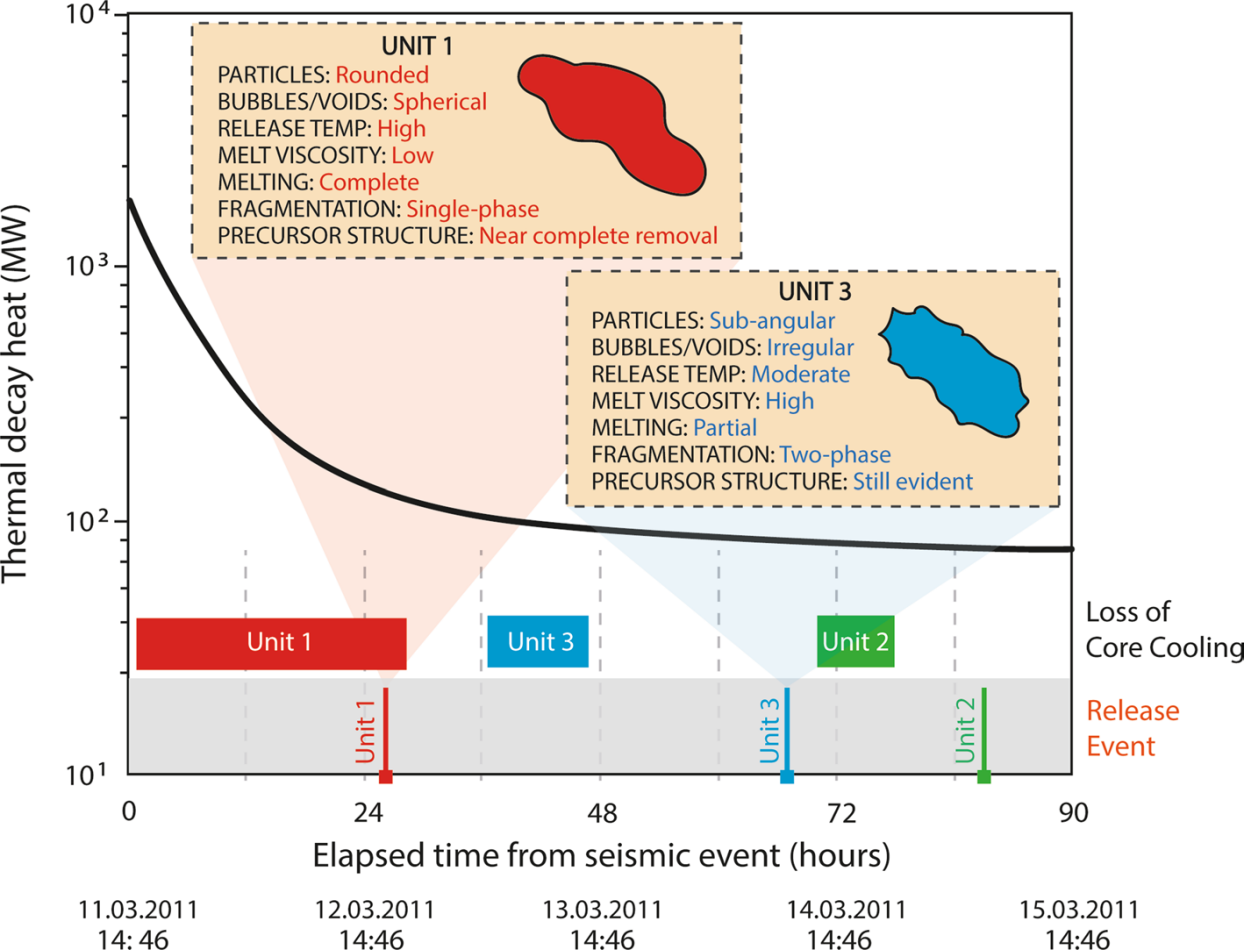
Schematic representation of reactor Unit 1 and Unit 3 particulate formation chronologies, alongside each suites resultant structural (internal and external) characteristics, and as observed during this study, the invoked conditions of formation.

We invoke the most likely explanation for the rounder shapes of Unit 1 particulates, as well as the more spherical bubbles within them, in comparison to Unit 3 particles, is that the molten precursor fragments that formed Unit 1 silicate particles were hotter and therefore the melt was considerably less viscous—supported by the known cooling chronologies of both reactors (Fig. 1), and as shown diagrammatically within Fig. 9. Over sufficient durations and in the absence of shear (deformational) stresses, surface tension will result in both the overall form of molten particulates and their internal bubbles becoming spherical because this minimizes the total surface area of gas–melt interfaces^44^. This rounding is resisted by the viscosity of the melt, with the characteristic timescale for rounding (τ_round_) given by^45^:

$$ \tau_{round} = \frac{\eta r}{\Gamma } $$

where Γ is surface tension (in the order of 10^–1^ N/m), η is the melt viscosity, and r is the radius of curvature of the interface (i.e. the radius of the particle or bubble). From this equation, the rounding of particles and internal bubbles can be seen as occurring faster if they are (i) smaller, and (ii) if the melt is less viscous. The sensitivity of the melt viscosity to temperature therefore means that the temperature of the ejected melt fragment could have the dominant control on how much rounding occurs before it is quenched, passing through the glass transition, forming a solid. Furthering the complimentary geological example of a dacite, discussed above, with r = 100 μm and Γ = 0.1 N/m; a τ_round_ of 0.006 s is observed at 1800 °C (η = 6 Pa s), in contrast to a τ_round_ of 9 s at 1200 °C (η = 9 × 10^3^ Pa s).

In contrast to the Unit 1 particles, the Unit 3 particle (Fig. 4e,f) has a highly complex fragmental core including glassy domains containing irregular-shaped bubbles alongside a component of preferential alignment; all hosted in a homogeneous glass. This preferred aligned of bubbles in the void-rich core domains could be a result of shearing of the bubble-bearing melt, however, we invoke a simpler explanation for this structure as representing a relic of the original orientation of the precursor glass fibers (and interstitial gas) within the parent material. The sintering of the glass fibers, when heated, would leave irregular bubbles with a preferred orientation parallel to the fiber, which, if it were not sufficient hot for sufficiently long for surface tension to round the bubble, could be preserved on re-cooling to glass. As such pieces of glass with irregular aligned bubbles exist alongside fragments of material with higher degrees of X-ray attenuation, we infer relatively cool temperatures of such core fragments. However, the glass around the core is homogeneous (Fig. 4e,f) and was therefore completely molten (and hotter, if the same chemistry as the particulates core material) when it entrained the core fragments. This homogeneous glass appears bubble-free, however, the semi-circular curved margin/surface located at the upper right-most extent in Fig. 4f could represent the margin of a bubble.

The sharp, angular margins of this homogeneous outer glassy region indicate brittle fracture. We suggest that this fracturing results from thermal stresses in the glass when these (originally larger) Unit 3 particles cooled, and that it corresponds to a secondary fragmentation stage after the primary (initial) event that generated the particle material—a subsequent episode not associated with the Unit 1 particulates. We do not have data to precisely constrain whether the primary fragmentation was brittle or fluidal (inertial) for any of the particles. Lower viscosities (higher-temperatures) are conducive to fluidal breakup, but even the hot melt of Unit 1 could fragment in a brittle manner if the strain rates were large enough^46^. Although the Unit 1 particles have fluidal shapes, this does not necessarily mean the fragmentation was fluidal, as surface tension can cause rounding of particles (and internal voids) while still hot.

Within this work, we investigated the composition and, for the first time, the structure of a suite of particulates invoked to have been derived from the FDNPP reactor Unit 3—contrasting their form and composition to those released from reactor Unit 1, as illustrated in Fig. 9. The structure of this particulate is a principal driver in its environmental stability/susceptibility to physical breakdown as well as elucidating as to the form and characteristics of the large inventory of material remaining within each reactors PCV—knowledge which will be invaluable in underpinning the future debris retrieval activities across the FDNPP site.

Of the two particulate types, we invoke the angular shape of the Unit 3 particles would be more susceptible to mechanical abrasion and attritional erosion of its sharp apices—resulting in its fragmentation and dispersion as finer-grained radioactive sub-particulates. However, the highly round and smoothed Unit 1 particles would be increasingly resistant to such mechanical breakdown. With respect to the environmental dispersion and transport of particulates, again drawing analogues to geological scenarios^47^, we deduce that as the associated drag forces (through the air, as a ballistic particle), particle free-fall (at terminal velocity) and the resultant distribution are dominated by the particle size—with secondary, and less substantial factors, being both the density (influenced by bubble content) and shape of the particulates, that both suites of material are to be observed at similar radial distances away from the FDNPP. As there likely occurs a location dependent particle size gradation with increased radial distance from the FDNPP, there would exist a finning of the particle (grain) size (alongside a corresponding increase in particle size towards the site) until further size reductions of the material are no longer observed. Such a lower-bound in particle size is attributed to the terminal velocity of the particles being less than the entraining flows turbulence velocity. Therefore, such fragmental particulate would not be encountered beyond a specific, and as yet undetermined, distance from the release center—a function of the transporting plume velocity.

As a result of these structural characteristics, we invoke that while the particulate derived from reactor Unit 1 would be largely stable and of low susceptibility to mechanical breakdown under surface environmental conditions (supported by their well-rounded form); contrasting behavior is to be associated with material from reactor Unit 3. Demonstrated by the fractured exterior surfaces, the non-spherical (and strength affording) bubbles and significant internal stresses, we identify such particulate as being highly likely to further fragment into smaller sub-particulates—with implications for subsequent radiological contamination migration. Such disparities in material brittleness and mechanical strength are additional considerations that should be evaluated when debris retrieval and reactor decommissioning operations are conducted at either reactor, where both particulates and residues of the precursor material are to be encountered. In the Fukushima case, it is hereby invoked that the angular silicate material from reactor Unit 3 represents a greater particulate generation hazard than the rounder and smoother, and so more mechanically resilient Unit 1 particles, which we attribute to the higher temperature of Unit 1 material when released, which allowed it to round before quenching to glass. During soon-to-commence decommissioning and dismantling operations within the damaged PCV structures of both reactors Unit 1 and 3 (as well as Unit 2) to remove the ‘slumped’ Corium and MCCI materials, by translating properties from the aforementioned magmatic system, we foresee that while the debris of Unit 1 will be significantly more challenging to remove via the proposed mechanical milling and scouring methods^48^, the radioactive dust production will be minimal, with material removal occurring via ‘as-cut’ volumes. In contrast, the inherent brittleness and associated high particulate generation potential of the angular material from reactor Unit 3 will likely result in the need for an alternate removal strategy be identified to mitigate against the significant aerosol generation hazard. However, although an issue for particulate formation, unlike the mechanically resilient fuel debris of reactor Unit 1, the reactor Unit 3 material is likely to be much more easily removed from within the confines of the PCV.

## Materials and methods

### Sample collection and preparation

Bulk sediment, road debris and dust, as well as fabric/cloth samples determined in the field (using handheld a radiation detector) to exhibit elevated levels of radioactivity were collected from sampling sites at 37.4379° N, 141.0222° E (CF designation), 37.4075° N, 141.0272° E (OH-02 designation) and 37.40° N, 141.02° E (OH-06 designation), both within 5 km of the site and to the north-west and south-west of the FDNPP, respectively. The material collected in each instance comprised between 10 and 150 g of sample and was selected due to its undisturbed nature and isolated position, where the potential for resuspension and re-contamination from other sources (e.g. roads and industrial processes) was determined to be minimal. Similar source material was formerly studied in the works of Ono et al.^33^, Satou et al.^25^ and Martin et al.^30^. An established imaging plate (IP) methodology was used to isolate the radioactive particles from an enclosing matrix^49^, using a BAS-SR 2025 (FUJIFILM Corporation) digital radiography scanner, with the material containing the microparticles having been prior screened using a wet separation method, based upon their differential density^50^. Through the multi-stage process comprising repeated autoradiography (with 5-min exposures to highlight only the radioactive particulates, while reducing the background intensity from the surrounding bulk) and sample division steps, the sub-mm particles that induced a localized blackening of the IP film were identified and removed using a manual micro-manipulation setup (AP-xy-01, Micro Support Corporation). Post-removal, the particulate was placed onto a small piece of adhesive carbon tape for subsequent initial scanning electron microscope (SEM)/energy dispersive spectroscopy (EDS) analysis and gamma-ray spectroscopy measurements. Following this surface characterisation, the radioactive particulate was then removed from the tape and fully enclosed within a double layer of adhesive Kapton film (DuPont Ltd.).

### Scanning electron microscopy (SEM) and energy dispersive spectroscopy (EDS)

Surface examination and compositional quantification of each particulate sample was performed using a Zeiss SIGMA Variable Pressure (VP) Scanning Electron Microscope (SEM) with complementary Octane Plus Si-drift characteristic Energy Dispersive Spectroscopy (EDS) X-ray detector from EDAX (AMETEK Ltd.). Each sample, attached to the adhesive carbon tape, was mounted onto a standard SEM pin-stub for analysis. During both imaging and EDS elemental analysis, a consistent 25 kV accelerating voltage, 120 µm aperture and 9 mm working distance were employed—all in the machines ‘High Current’ mode to enhance the on-sample current. The negate against the detrimental influence of sample charging resulting from the non-conductive nature of the material examined, the VP function of the instrument was used—maintaining a constant flow of nitrogen gas over the particle to rapidly remove electron-induced charge build-up. All bulk EDS spectrum were obtained over a user-defined region comprising no less than 85% of the particles surface, with an acquisition period of no less than 200 s (true detector live time) to derive sufficient peak intensity appropriate for deriving quantifiable results. Both control of the detector and the subsequent processing of results were performed using the instruments EDAX TEAM software.

### Gamma-ray spectroscopy

For attribution of each of the particulates to their reactor Unit source, quantitative gamma-ray spectroscopy results were derived by placing each sub-mm sample (enclosed inside Kapton film) within a cryogenically cooled High Purity Germanium (HPGe) detector; GEM40-76, (ORTEC, USA) with associated multi-channel analyzer (MCA). Prior to each measurement, a calibration of the detectors efficiency and emission peak energy position was undertaken using a ‘multi-source’ reference standard from the Japan Radioisotope Association (JRA). In order to ensure appropriate signal-to-noise in each spectrum, the counting time for each sample was varied such that a total of 10,000 counts of ^134^Cs (net count) was recorded.

### Laboratory X-ray tomography

Prior to the limited synchrotron radiation (SR) beamtime analysis, pre-screening of all Kapton enclosed particulates was performed using complementary laboratory X-ray tomography (XRT) to identify regions appropriate for subsequent (synchrotron radiation) analysis. Whereas the synchrotron is capable of delivering significantly more X-ray photons onto the sample, the laboratory XRT is able to obtain finer absorption contrast pixel (voxel) spatial resolutions—on the order of 50 nm, opposed to the micron-scale pixels attainable at the synchrotron, albeit at significantly slower acquisition rates. While the laboratory XRT provides superior tomographic spatial resolutions, unlike SR however, it is not possible to obtain spatially derived compositional information (via X-ray fluorescence) using the laboratory setup—with such information obtainable only using synchrotron radiation.

For the X-ray analysis, each Kapton enclosed particle was mounted onto the tip of a stainless-steel support pin attached to a magnetic base, which was then installed within the instruments multi-axis (+ rotation) stage. A Zeiss Xradia 520 Versa μXRT was used to obtain 3D tomographic data of the samples, which operated at 80 kV with no additional filtering. Collection was obtained using either the 20 × or 40 × optical magnification, depending on the sample size, with the generated image collected on an ANDOR low-light camera.

Statistical analysis and quantification of the internal void diameters and associated aspect ratio within the particles was performed on the TIFF slices generated from each of the tomographic reconstructions. The opensource ImageJ software suite and image processing plug-ins^51^ were used to determine; (i) the proportion of each slice that was composed of (black) space and therefore a void, alongside (ii) the aspect ratio of each such void—defined as the ratio of the longest to the shortest axis^52^, shown schematically in Figure S3.

### Synchrotron radiation X-ray analysis

The SR analysis was undertaken on a subset of particulates identified as possessing representative features (both surface and internal), elemental composition and isotopic activities from both groups (CF-xx and OH-xx), at the Diamond Light Source synchrotron (Harwell, UK). In all instances, the analysis utilized the same Kapton sample enclosures as used for laboratory X-ray studies to contain the sub-mm particulates. Two of Diamond’s beamlines were used in this study; I13-1 (coherence imaging) and I18 (micro-focus spectroscopy)—with reactor Unit 1 materials examined on I13-1, and reactor Unit 3 samples analysed using the I18 beamline.

#### I13-1

As the longest beamline at the facility, in order to maintain special coherence through the vertically aligned experimental optics, a distance of 250 m exists between the insertion device (canted undulator) and the sample^53^—with the beamline capable of exploiting X-rays with energies of 4–23 keV (although the maximum energy of the XRF spectra was 18 keV to avoid the large peak at 19 keV, representing the incident beam) with an on-sample flux of 10^9^ photons/s.

To derive the 3D volumetric XRF compositional analysis of the particle, following standard beamline focusing optics, the broad X-ray beam was subsequently focused through a 5 μm pinhole to produce a corresponding diameter on-sample beam spot. At each 4.5° rotational angle of the 3D XRF scan (over 180°), the sample was translated with respect to the centered beam in a raster grid of 40 × 20 steps—with each step of 2.5 μm, therefore yielding a field of view/scan volume of 100 μm × 50 μm comprising 32,000 spectrum containing measurement points. A corresponding pixel size of approximately 2.5 µm was hence produced. At each scan position, the XRF spectrum was acquired using a Vortex single channel silicon drift X-ray detector placed level with the height of the sample, normal to the beam path. From the computed stacks of fluorescence projections, the three-dimensional volume for each element was reconstructed using the ordered-subset penalized maximum likelihood algorithm, with weighted linear and quadratic penalty algorithms in the TomoPy framework^54^, with an iterative algorithm simultaneously used to correct for the degree of sample self-absorption that occurred (employing prior synchrotron tomography results). The reconstructed images were produced using ImageJ and Python software platforms.

#### I18

A comparable optical setup was also used on the I18 beamline, with the beamline attaining a comparable incident X-ray energy range of 3–22 keV (although a maximum incident energy of 19 keV was similarly used on I18), using a cryogenically cooled Si-111 monochromator for beam energy selection^55^. Resulting from the beamlines closer position to the insertion device and the absence of necessity to maintain special coherence of the beam, a greater on-sample photon flux is achieved, with a full beam size flux at 10 keV of 10^12^ photons/s—focused to a 2 µm × 2 µm incident beam. To fully utilise this more highly focused beam, the Kapton™ encapsulated particle sample was also rastered in 2 µm translational steps, therefore, resulting in a 2 µm × 2 µm spatial (pixel) resolution.

In a methodology contrasting with that of I13-1, to derive the 3D XRF reconstructions of the particle on the I18 beamline, a series of XRF tomographic ‘slices’ (each 2 µm in thickness) of the particulate were obtained by translating/rastering the sample through the beam path—yielding a 2 µm × 2 µm 2D scan, with signal generated from the entire sectional volume. The sample was then rotated by 0.5° and a subsequent 2D raster scan of the sample obtained. This was repeated through 0°–180° of sample rotation before the sample height was changed and the 2D rastering and rotation sequence repeated to yield a total of nine sections each separated by 23 µm. Computation of these 2D sections into 3D volumetric data for analysis and visualisation was performed using the Diamond Light Sources tomographic reconstruction software ‘Savu’^56^ and ‘Dawn’ processing suite^57^. Derivation of the entire particle (bulk) XRF spectrum was obtained through summing the fluorescence signals associated with each tomographic slice; processed and peak fitted using the Python Multichannel Analyzer (PyMCA) software suite.

#### Compositional coincidence

Determination of the qualitative degree of elemental spatial correlation within particulate samples was derived using a custom Python analysis script to yield values of the R-Squared (R^2^) correlation using the SR-µ-XRF results. From the cross-sectional slices derived for each element (using element-specific energy windows within the XRF spectra) a pixel-by-pixel comparison of the volumetrically normalised signal intensities was performed—with the results combined to yield an inter-element coincidence value.

## Supplementary Information

Supplementary Figures.

## Data Availability

Data contributing to this manuscript is available at 10.17632/wg3dn35ssw.1.
