# Supplementary material for: Structural and compositional characteristics of Fukushima release particulate material from Units 1 and 3 elucidates release mechanisms, accident chronology and future decommissioning strategy
Source: Sci Rep. 2020 Dec 16;10:22056. doi: 10.1038/s41598-020-79169-2 (PMC7744541; doi:10.1038/s41598-020-79169-2)
Supplement: Supplementary file 1 — Supplementary Figures. [file 41598_2020_79169_MOESM1_ESM.docx]

Supporting Information for:

Structural and compositional characteristics of Fukushima particulate material from Units 1 and 3 elucidates release mechanisms, accident chronology and future decommissioning strategy

Peter G. Martin ^1^ *, Christopher P. Jones ^1^, Stuart Barlett ^2^, Konstantin Ignatyev ^2^,

Dave Megson-Smith ^1^, Yukihiko Satou ^3^, Silvia Cipiccia ^2^, Darren J. Batey ^2^, Christoph Rau ^2^,

Keisuke Sueki ^4^, Tatsuya Ishii ^4^, Junya Igarashi ^5^, Kazuhiko Ninomiya ^5^, Atsushi Shinohara ^5^,

Alison Rust ^6^, Thomas B. Scott ^1^

^1^ Interface Analysis Centre, School of Physics, University of Bristol, Bristol, BS8 1TL, UK

^2^ Diamond Light Source, Harwell Science and Innovation Campus, Didcot, Oxfordshire, OX11 0DE, UK.

^3^ Collaborative Laboratories for Advanced Decommissioning Science (CLADS), Japan Atomic Energy Agency (JAEA), Tomioka-Machi, Futaba-gun, Fukushima 979-1151, Japan.

^4^ Graduate School of Pure and Applied Sciences, University of Tsukuba, 1-1-1 Tennodai, Tsukuba, Ibaraki 305-8577 Japan.

^5^ Graduate School of Science, Osaka University, 1-1 Machikaneyama, Toyonaka, Osaka, 560-0043, Japan.

^6^ School of Earth Sciences, Wills Memorial Building, University of Bristol, Bristol, BS8 1RJ, UK.

** corresponding author*

*Email: peter.martin@bristol.ac.uk Telephone: +44 (0) 117 33 17684*


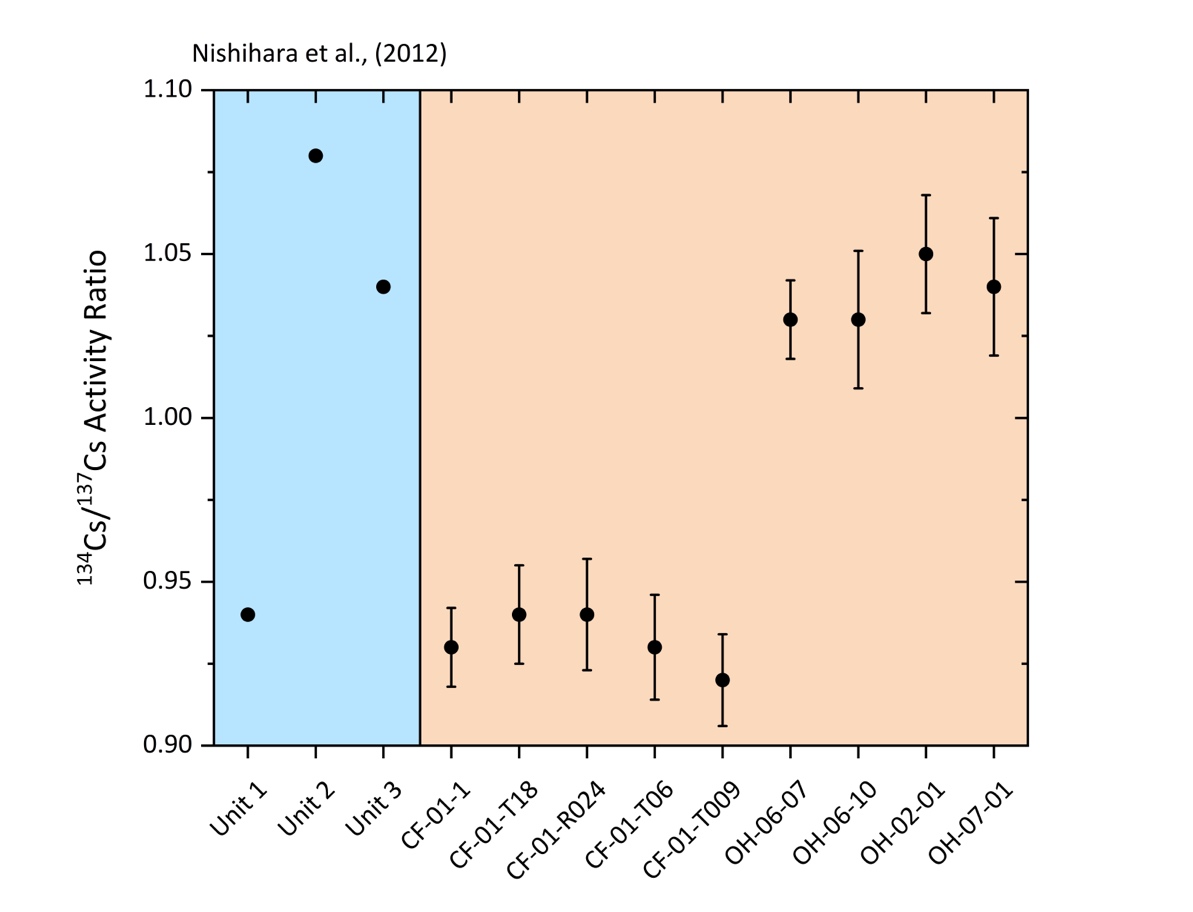


***Figure S1.*** *Complete reactor core and fuel storage pond (FSP) inventory estimates for both Cs activity (^134^Cs/^137^Cs) and isotope ratio (^135^Cs/^137^Cs) at the time of release (decay corrected to 11/03/2011). Values from the ORIGEN calculations undertaken by ﻿Nishihara et al.*^1^

*^1^ Nishihara, K., Iwamoto, H. & Suyama, K. Estimation of fuel compositions in Fukushima-Daiichi nuclear power plant (in Japanese). JAEA 2012-018 (2012).*


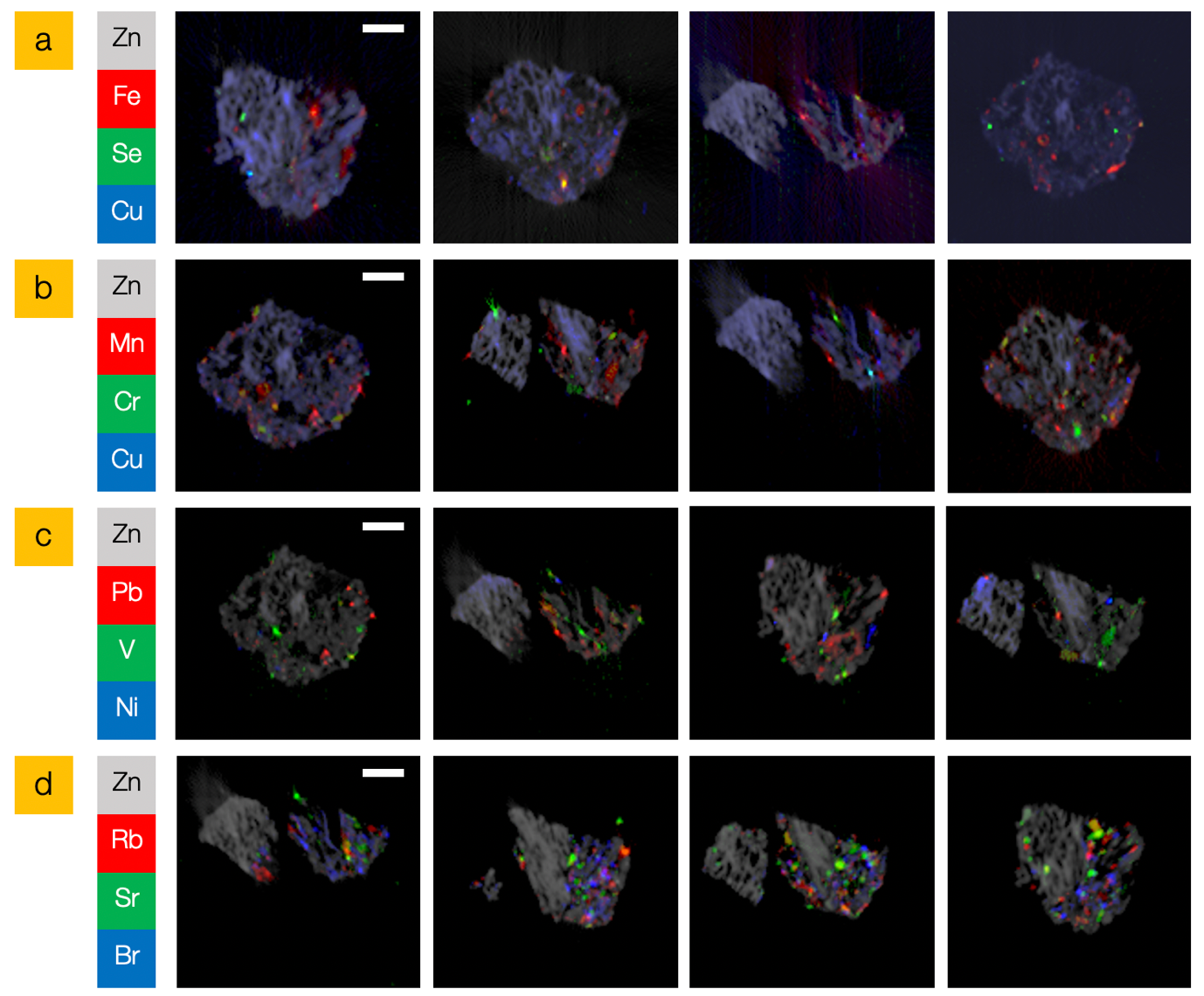


***Figure S2.*** *SR-µ-XRF longitudinal sections obtained at varying section heights through FDNPP Unit 3 derived particle OH-06-07, comprising Grey+RGB compositional species information, for* ***(a)*** *Zn+Fe+Se+Cu,* ***(b)*** *Zn+Mn+Cr+Cu,* ***(c)*** *Zn+Pb+V+Ni, and* ***(d)*** *Zn+Rb+Sr+Br.*


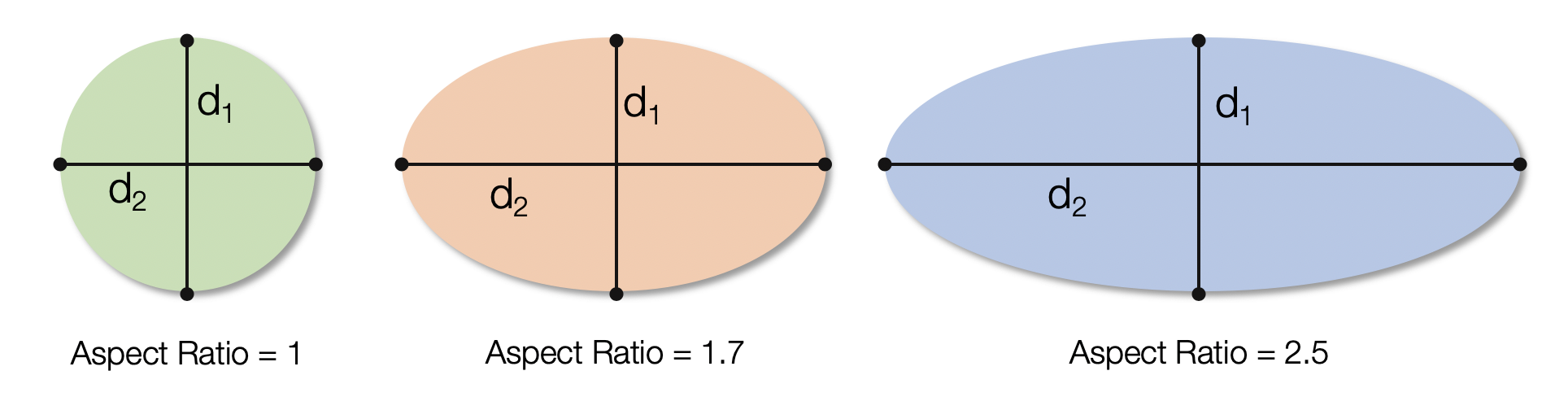


***Figure S3.*** *Schematic representation of the determination of void aspect ratio as determined by the ratio of orthogonal axis (d_1_ and d_2_), as defined in Bullard and Garboczi (2013)*^2^*.*

^2^ Bullard, J. W. & Garboczi, E. J. Defining shape measures for 3D star-shaped particles: Sphericity, roundness, and dimensions. *Powder Technol.* 249, 241–252 (2013).
